# Matters of Gender and Social Disparities Regarding Postnatal Care Use Among Nepalese Women: A Cross-Sectional Study in Morang District

**DOI:** 10.1089/heq.2022.0186

**Published:** 2023-05-19

**Authors:** Rakchya Amatya, Mathuros Tipayamongkholgul, Nawarat Suwannapong, Siriwan Tangjitgamol

**Affiliations:** ^1^ GTA Foundation, Lalitpur, Nepal.; ^2^ Master of Public Health Program, Faculty of Public Health, Mahidol University, Bangkok, Thailand.; ^3^ Department of Epidemiology, Faculty of Public Health, Mahidol University, Bangkok, Thailand.; ^4^ Faculty of Public Health, Mahidol University, Bangkok, Thailand.; ^5^ Department of Obstetrics and Gynaecology, Faculty of Medicine, Vajira Hospital, Navamindradhiraj University, Bangkok, Thailand.

**Keywords:** Nepal, postnatal care, social disparity, women's autonomy

## Abstract

**Objective:**

The study compares the uses of postnatal care (PNC) and women's autonomy gradients across social caste and used intersectionality concepts to estimate odds ratio of women's autonomy and social caste on complete PNC.

**Methods:**

A community-based cross-sectional study among 600 women aged 15–49 years who had at least one child younger than the age of 2 years in Morang District, Nepal, was conducted from April to July 2019. PNC, women's autonomy (decision-making power, freedom of movement, and control over finances) and social caste were collected by both methods. Multivariable logistic regressions were used to determine associations between women's autonomy, social caste, and complete PNC.

**Results:**

Complete PNC totaled 13.5% of respondents. About one-fourth of respondents reported poor overall autonomy; however, non-Dalit demonstrated higher autonomy than Dalit. Non-Dalit exhibited greater odds of complete PNC by four times. Women exhibited high women's autonomy in decision-making power, control over finance, and freedom of movement and have greater odds of complete PNC than low autonomy by 17, 3, and 7 times, respectively.

**Conclusion:**

The study raises awareness of intersectionality (gender and social caste), relating to maternal health in caste-based system countries. To improve maternal health outcomes, health care personnel should identify and systematically address barriers that women of lower-caste membership face and offer these women appropriate advice or resources to obtain care. A multilevel change program that involves different actors like husbands and community leaders is needed for improving women's autonomy and lessening stigmatized perceptions, attitudes, or practices toward non-Dalit caste-members.

## Introduction

Lessening the maternal mortality ratio (MMR) to below 70 per 100,000 live births by 2030 is likely a great challenge to countries with a low Human Development Index (HDI), including Nepal^1^ where MMR is about 40 times higher than others.^2^ Nepal has MMR of 186 per 100,000 live births.^2^ Over one-third of maternal deaths (34%) in Nepal occurred during the postpartum period and the majority involved postpartum hemorrhage,^3^ similar to other low HDI countries.^4,5^ To improve this situation, the Nepal Ministry of Health and Population (MoHP) recommends that women utilize postnatal care (PNC) at least three times, that is, within 24 h, 3, and 7 days postpartum.^6^ Unsuccessful implementation was reported by the latest national demographic and health survey at about 42.0% of women not using PNC^7^ and completing three PNC visits decreased from 19.0% to 16.0% in 2016 and 2018, respectively.^6^

Low PNC utilization is crucially influenced by social determinants, in which women are born, grow, work, live, and age.^8,9^ Such social conditions can structurally influence lived experiences of women, constrain resources and social opportunities that later position women into disadvantage and social stratification.^9^ Studies in Ethiopia,^10^ India,^11^ and United Kingdom^8^ emphasized disparity of maternal health and maternal health service use attributable to social determinants. Among social determinants, caste is a hereditary social stratification that determines settlement condition, marriage irrespective of faith practices of individual.^12^ Individuals in disadvantage caste groups are critically prone to disparities in health and service accessibility.^12,13^

Consistently, maternal care utilization among women in disadvantage caste group in central Asia countries were lower utilization of maternal care^14,15^ despite the free of charge service.^13^ Women who are born in low-caste group likely have poor accessibility^16–18^ to maternal health service due to geography and financial constraint, social discrimination, husband's education, and lack of autonomy.^8,19,20^

Women's autonomy is an ability through which women can act upon basic aspects of their life that affects their health and well-being.^19,20^ As a country with a patriarchal system, women in Nepal face an unjust imbalance of power and women's related barriers.^21^ Gender gaps can be found in education, income opportunities, legal rights, and health care accessibility. Nepalese women have lower autonomy than men in self-health care decision-making.^22^

In addition, Nepal maintains a caste-based hierarchical system, in which Brahmin/Chhetri is at the topmost of the hierarchy (advantaged caste), whereas Dalits are at the bottom (disadvantaged caste).^23^ Dalits have faced a complicated interconnection of historical and social biases. They generally lack human dignity and justice and may have the inability to access health services.^24,25^ Related studies in Nepal reported the highest MMR and lowest use of PNC among Dalits.^3,23^

Caste-based discrimination and gender inequity may lessen health care use among Dalit women and cause poor health outcomes among them. Effects of interconnection between social classes, ethnic, caste, and gender on women's health have widely raised awareness among public health professional.^26–28^ To examine insightful explanation of women health inequity, intersectionality concepts has become a crucial framework to study women's disadvantage.^26,27^

The Morang District lies in the Terai Region of eastern Nepal, and is ethnically diverse.^29^ The district has reported a lower percentage of PNC use than the national average.^6,30^ Social caste and gender inequity may play a crucial factor in PNC use in the area involving diverse cultures such as the Morang. We aimed to examine the relationship between women's autonomy, social caste, and complete PNC. The findings could assist health personnel to gain a better understanding of low PNC, and provide insights to improve PNC in countries with patriarchal and caste-based systems.

## Methods

### Study design and settings

The community-based cross-sectional study was conducted in one urban (Rangeli) and one rural (Kanepokhari) in the Morang District from April 2019 to July 2019. Morang District lies in Province No. 1 and divides into nine municipalities and eight rural areas. Rangeli and Kanepokhari were purposively selected because of the availability of the large proportion of the Dalit group who reside in these regions. Rangeli is further divided into nine wards whereas Kanepokhari is divided into seven wards.

### Study participants

#### Study population and Sample

Women aged 15–49 years who have at least one child younger than the age of 2 years living in two selected areas of Morang District were included. Exclusion criteria included (1) hearing impairment or inability to speak and (2) being hospitalized longer than 24 h after delivery of the last child due to complications or in need of treatment.

#### Sample size and sampling procedure

The adequate sample size was calculated by two population proportions formula^31^ with 5% of Type I (5%) and 20% of Type II error. Due to the lack of PNC statistics among social caste, we used proportions of PNC within 2 days between rural (0.48) and urban areas (0.64) from the national survey^7^ to calculate the sample size and yielded 300 subjects per group.

Two-stage stratified probability proportional to size sampling^32^ was used to select 300 women from three wards of the Rangeli and Kanepokhari areas. The first stage, we select three wards from each area, and then used simple random sampling to select 100 eligible women from each selected ward's rosters of women which collected by Female Community Health Volunteers (FCHVs). By this sampling technique, each individual in the population possessed the same probability of being sampled.^32^ All 600 selected eligible women were informed of the research information and face-to-face interviews were commenced after the women agreed and signed the consent forms.

### Research instrument

To collect quantitative data, we used the face-to-face interview-structured questionnaire. The dependent variable was PNC use. Three questions with a two-scale response (yes, no) were used to assess complete PNC, that is, use within 24 h of delivery, use on the third day, and use on the seventh day after childbirth. Positive responses to all three questions were considered complete PNC.

Independent variables included social caste which was classified in two categories: (1) Dalit or (2) non-Dalit, including Brahmin/Chhetri, Janajati, and other castes, and women's autonomy. Women's autonomy was measured through three dimensions, including control over finances, freedom of movement, and decision-making power using a 10-item structured questionnaire that was developed Bloom et al.^33^ It used an anchored 3-response scale, ranging from No/never, Yes, sometimes, and Yes, always.

Control over finances was assessed through respondents' answers to a series of three different circumstances: (1) regular access to money, (2) savings for personal use, and (3) spending money without consulting anyone.

Freedom of movement was assessed through respondents' freedom to go out solely on four different conditions: (1) going to the market, (2) taking a child to the doctor, (3) visiting a doctor for their health care, and (4) visiting natal kin/family.

Decision-making power was assessed through respondent's power on three different types of household decisions: (1) going out of the house without seeking permission, (2) deciding small matters such as what to cook, and (3) deciding larger matters such as schooling for children divided as family members, jointly with others or solely decided.

Previous studies found that accessibility to a health facility, age at the last childbirth of the respondents, highest education of respondent, and highest education of respondent's husband were associated with PNC.^8,10,16–18^ Therefore, these factors were adjusted to reveal a less biased odds ratio (OR) of social caste and women's autonomy on complete PNC.

Accessibility to health facility comprised four domains: (1) geographical accessibility, (2) financial accessibility, (3) cultural accessibility, and (4) functional accessibility. Questions were open-ended and close-ended. For close-ended questions, there were three responses: “Yes, always=3,” “Yes, sometimes=2,” “No, never=1,” and two responses: “Yes=2,” “No=1.”

The Cronbach's alpha coefficient for women's autonomy was 0.867. The total scores for each part were classified into three groups. Of the total score for women's autonomy, more than or equal to 80.0% was considered high, 60.0–79.0% was moderate, and less than 60.0% was needed to improve.

The research assistants were recruited from local health assistants (FCHVs). They could fluently speak both the local dialect and Nepali and had assisted in collecting data in previous surveys conducted in Nepal. They passed a 1-day training to understand study objectives, the questionnaire.

This study was approved by the Ethics Committee of the Faculty of Public Health, Mahidol University (MUPH 2019-048), and the Nepal Health Research Council (Ref. No: 2778). Informed consent was obtained from all participants. For illiterates, the first author read the content on the consented form, and the respondents stamped thumbprints on the consent form, and, for minors, verbal permission from the respondents' legal guardians to interview the respondents aged younger than 16 (3 respondents) years were obtained, which was approved by the Nepal Health Research Council. All methods were carried out in accordance with relevant guidelines and regulations.

### Data analysis

Percentage, frequency, mean, and standard deviation were used to compare general characteristics, women's autonomy, and use of PNC by social caste. A *p*-value <0.05 was considered statistically significant. To estimate ORs of women's autonomy and social caste on complete PNC, we first conducted binary logistic regression to calculate OR of social caste on complete PNC. Second, we used multivariable logistic regression to calculate OR of social caste and each domain of women's autonomy on complete PNC. Finally, we conducted multivariable logistic regression to assess less bias OR of social caste and women's autonomy for complete PNC after controlling effects of geographical accessibility, financial accessibility, cultural accessibility, functional accessibility, age at birth of the last child, highest education of respondent, and highest education of respondent's. ORs and 95% confidence intervals (CIs) were calculated by binary logistic regression.

## Results

### Use of PNC among respondents

**FIG. 1.**
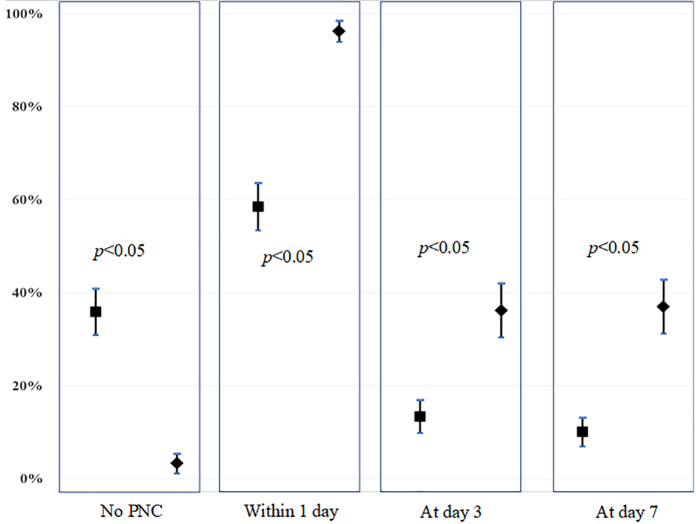
Use of PNC among respondents by social caste. PNC, postnatal care.

All selected women (600 respondents) voluntarily participated in the study (response rate=100%). Of 600 respondents, 50.7% of 335 Dalit respondents and 49.1% of 265 non-Dalit respondents resided in Kanepokhari (Table 1). Of the 600 respondents, only 13.5% reported complete PNC. PNC use at each recommended time differed between social caste; non-Dalit respondents reported to use PNC statistically higher than Dalit respondents at each timing (Fig. 1). The first visit which occurred 1 day postpartum was statistically higher among non-Dalit respondents (96%) compared to the Dalit respondents (58%), however, the percentage of PNC use in both groups declined by 50% in the following two timings (at day 3 and 7). Moreover, about 36% of Dalit respondents did not use PNC after delivery, while only 4% of non-Dalit women did not (Fig. 1).

**Table 1.** Characteristics of Respondents

Characteristic	Total (***n***=600)	Dalit (***n***=335)	Non-Dalit (***n***=265)	
	***n*** (%)	***n*** (%)	***n*** (%)	***p***
Areas				0.681
Rangeli	300 (50.0)	165 (49.3)	135 (50.9)	
Kanepokhari	300 (50.0)	265 (50.7)	130 (49.1)	
Age at birth of last child (years)				
15–19	85 (14.2)	57 (17.0)	28 (10.6)	0.034
20–24	242 (40.3)	128 (38.2)	114 (43.0)	
25–29	186 (31.0)	94 (28.1)	92 (34.7)	
30–34	57 (9.5)	35 (10.4)	22 (8.3)	
≥35	30 (5.0)	21 (6.3)	9 (3.4)	
Mean±SD	24.4±4.9	24.4±5.2	24.3±4.4	
Min–max	15–41	15–41	16–39	
Highest education of respondents				
Illiterate	145 (24.2)	128 (38.2)	17 (6.4)	<0.001^a^
Primary	132 (22.0)	104 (31.0)	28 (10.6)	
Secondary	89 (14.8)	42 (12.5)	47 (17.7)	
High school	215 (35.8)	61 (18.3)	154 (58.1)	
Bachelor degree and above	19 (3.2)	—	19 (7.2)	
Highest education of respondent's husband				
Illiterate	101 (16.9)	91 (27.1)	10 (3.8)	<0.001
Primary	119 (19.8)	92 (27.5)	27 (10.2)	
Secondary	114 (19.0)	70 (20.9)	44 (16.6)	
High school	237 (39.5)	80 (23.9)	157 (59.2)	
Bachelor degree and above	29 (4.8)	2 (0.6)	27 (10.2)	
Average household monthly income (Nepalese Rupee)				
<6000	258 (43.0)	207 (61.8)	51 (19.2)	<0.001
6000–10,000	206 (34.3)	93 (27.8)	113 (42.6)	
>10,000	136 (22.7)	35 (10.4)	101 (38.2)	
Median	6000	5000	10,000	
Min–max	1000–40,000	1000–30,000	1500–40,000	

1 USD=110.22 Nepalese Rupee.

^a^ Fisher's exact test.

SD, standard deviation.

### General characteristics of respondents

The study found that adolescent pregnancy among Dalit women was nearly twice as high as that among non-Dalit women (17.0% vs. 10.6%). A higher proportion of non-Dalit respondents along with their husbands had obtained high school education level. The majority of respondents in both groups were homemakers. The proportion of non-Dalit having monthly household income over 10,000 Nepalese Rupees was over three times higher than that in the Dalit group (38.2% vs. 10.4%) (Table 1).

### Women's autonomy among respondents

Women's autonomy among respondents differed significantly by social caste (*p*<0.001). Non-Dalit respondents were more likely to have higher autonomy. Likewise, a larger percentage of non-Dalit respondents had higher control over finances compared with Dalit respondents (20.0% vs. 6.5%), decision-making power (13.2% vs. 1.5%) and freedom of movement (37.0% vs. 16.4%) (Table 2).

**Table 2.** Women's Autonomy of Respondents by Social Caste

Women's autonomy	Total (***n***=600)	Dalit (***n***=335)	NonDalit (***n***=265)	
	***n*** (%)	***n*** (%)	***n*** (%)	***p***
Women's autonomy (overall)				
Poor	168 (28.0)	130 (38.8)	38 (14.4)	<0.001
Moderate	371 (61.8)	205 (61.2)	166 (62.6)	
High	61 (10.2)	—	61 (23.0)	
Control over finances domain				
Poor	330 (55.0)	219 (65.4)	111 (41.9)	<0.001
Moderate	195 (32.5)	94 (28.1)	101 (38.1)	
High	75 (12.5)	22 (6.5)	53 (20.0)	
Decision-making power domain				
Poor	304 (50.7)	234 (69.9)	70 (26.4)	<0.001
Moderate	256 (42.6)	96 (28.6)	160 (60.4)	
High	40 (6.7)	5 (1.5)	35 (13.2)	
Freedom of movement domain				
Poor	105 (17.5)	81 (24.2)	24 (9.0)	<0.001
Moderate	342 (57.0)	199 (59.4)	143 (54.0)	
High	153 (25.5)	55 (16.4)	98 (37.0)	

### Associated factors with complete PNC

Crude odds of complete PNC were higher among non-Dalit than Dalit by eight times (crude OR=8.550; 95% CI 4.603–15.88). After adding women autonomy in to the model, adjusted odds of complete PNC among non-Dalit was 3.276 (95% CI 1.606–6.684), among high level of decision-making power (adjusted odds ratio [AOR]=15.431; 95% CI 4.935–48.247), high level of control over finances (AOR=2.102; 95% CI 0.827–5.343), and freedom of movement (AOR=5.396; 95% CI 1.474–19.785).

In our final model adjusting for geographical accessibility, financial accessibility, cultural accessibility, functional accessibility, age at birth of last child, highest educational level of respondent, and highest educational level of respondent's husband, we found that complete PNC assessments were higher among non-Dalit respondents (AOR=4.943; 95% CI 2.01–12.135), high level of decision-making power among respondents (AOR=17.841; 95% CI 4.792–66.418), control over finances among respondents (AOR=3.127; 95% CI 1.064–9.187), and freedom of movement (AOR=7.522; 95% CI 1.929–29.335) (Table 3).

**Table 3.** Odds Ratio and Adjusted Odds Ratio of Social Caste and Women Autonomy for Complete Postnatal Care

	Model 1				Model 2				Model 3			
		95% CI for AOR				95% CI for AOR				95% CI for AOR		
	Crude OR	Lower	Upper	***p***	AOR	Lower	Upper	***p***	AOR	Lower	Upper	***p***
Social caste												
NonDalit	8.550	4.603	15.881	<0.001	3.276	1.606	6.684	0.001	4.943	2.013	12.135	<0.001
Women autonomy												
Decision making power												
Low												
Moderate					2.010	0.971	4.163	0.060	2.215	0.997	4.919	0.051
High					15.431	4.935	48.247	<0.001	17.841	4.792	66.418	<0.001
Control over finance												
Low												
Moderate					0.923	0.453	1.879	0.824	1.001	0.472	2.124	0.998
High					2.102	0.827	5.343	0.118	3.127	1.064	9.187	0.038
Freedom of movement												
Low												
Moderate					1.301	0.367	4.609	0.684	1.403	0.386	5.096	0.607
High					5.396	1.474	19.758	0.011	7.522	1.929	29.335	0.004

Model 3 adjusted for geographical accessibility, financial accessibility, cultural accessibility, functional accessibility, age at birth of last child, highest educational level of respondent, highest educational level of respondent's husband.

95% CI, 95% confidence interval; AOR, adjusted odds ratio; OR, odds ratio.

## Discussion

The study revealed that the complete PNC use in the study area was far below the national target (50.0% in 2020)^6^ and complete PNC among Dalit women was lower than among non-Dalit women. This study found that the proportion of PNC use among Dalit women was lower than that reported in the national survey^12^ because the survey analyzed only single PNC visits within 2 days of childbirth.

This study emphasized the effects of the intersectionality of social caste and gender power disparity on complete PNC. Dalit women were less likely to comply with recommended PNC use compared with non-Dalit women. The reason behind this disparity may be the poor social and economic conditions of the Dalit group. In the study area, the Dalit were found to have obtained lower educational levels, earned less income, and been involved in more laborious work compared with the non-Dalit group. Similarly, women with low education levels and low wealth index were less likely to use maternal health services than women with high education levels and high wealth index in other studies in Benin,^34^ India,^11,15^ Nepal,^14,35^ Nigeria,^18,36^ and Pakistan.^14,17^ Both Dalit and non-Dalit women generally have low levels of autonomy, except for a slightly higher freedom of movement among non-Dalits.

Dalits may experience mobility restrictions because of socioeconomic disadvantage, poverty, illiteracy, and limited resources. In addition, Dalit women are more likely to be the victims of violence, sexual assault, and harassment, which restrict their freedom of movement and prevents them from traveling.^12–15,25,37,38^ The lower PNC use among Dalit women may also be related to poor self-awareness and lack of health information accessibility due to social bias. Many women in our study areas revealed that they were unaware of risky conditions and adverse events after delivery. They mentioned that PNC was unnecessary unless any severe conditions were present. Poor knowledge and household roles of women such as taking care of household members, parenting, and domestic responsibilities could lessen this awareness of PNC among Asian women.

Lack of awareness regarding PNC services and their importance was found to be a barrier to receiving these services among women in Ethiopia,^10,39^ Uganda, and Zambia.^40^ In addition, social isolation due to daily household chores among Nepalese women was also found to have negative effects on using maternal health care.^41,42^

Women's autonomy, including control over finances, was also found to be associated with complete PNC use. In the study area, most women were not allowed to spend money without consulting their husbands or other family members. Also, the majority could not set aside money for their personal use. These situations might hinder the women from visiting a health facility to receive PNC services. A positive association was reported between women's control over finances and use of maternal health services in other studies in Bangladesh,^41^ Nepal,^42,43^ and sub-Saharan African countries.^38,44^ This relationship could be supported by the fact that when women are given the power for controlling financial assets, they will have the ability to make choices and assess the costs and benefits of the available resources. As a result, she can use those resources in the most effective way.^45^

Women having the power to make decisions were more likely to use complete PNC services in this study. Other studies conducted in Afghanistan, Ethiopia, and Nepal also concluded that the decision-making power of women concerning their health, household purchases, and women's mobility was positively associated with maternal health service use.^46,47^ This could be further explained as women possessing the power to make their own decision can control their health care and decrease their risky behaviors.^48^ However, only a small proportion of the respondents usually decided all by themselves. The majority were not allowed to leave the house without permission. One woman during the data collection expressed her autonomy and explained her compulsion to follow her mother-in-law's advice and not receive any services she presumed were unnecessary.

Women's freedom of movement and complete PNC use were positively correlated. Having said that, some women in the study area were not allowed to solely visit the doctor as well as travel to places such as local market and natal home. This restricted mobility may limit their access to information and lower their decision-making power even concerning personal matters such as their health care. Furthermore, women's freedom of mobility, decision-making, and rights remain limited due to the prevalence of patriarchal systems in Nepal.^49^ The association of freedom of movement with maternal health use has also been reported in India^33–49^ and Bangladesh.^41^

### Strength and limitations

Data were collected among large samples to compare between Dalit and non-Dalit groups. Due to the cross-sectional nature of this study, the respondents reported their past behaviors which might have been influenced by remembering their experiences inaccurately or omitting details. However, women who had delivered their baby within the past 2 years were chosen to minimize any recall bias. Moreover, lack of women's autonomy, being a sensitive issue, could also have involved reporting bias. However, interviews were conducted privately and were designed to reduce this issue. Although this study used local translators, they were trained before collecting data to reduce information errors.

## Health Equity Implication

The study revealed that social caste plays an important role on postnatal health service utilization. This study also raises awareness on the intersectional effects of gender and caste on maternal health care in countries where caste systems are still legally present or socially reinforced. To improve maternal health outcomes, health care personnel should identify and systematically address barriers that women of lower caste membership face and offer these women appropriate advice or resources to obtain care. In addition, it is necessary to formulate a program that involves different actors like husbands, family members, and community leaders and impacts multilevel change for improving women's autonomy and also lessens the stigmatized perceptions, attitudes, or practices toward non-Dalit caste-members.

## Abbreviations Used

AOR: adjusted odds ratio
CI: confidence interval
FCHV: Female Community Health Volunteer
HDI: Human Development Index
OR: odds ratio
PNC: postnatal care
SD: standard deviation
